# Characterization of Bacterial and Fungal Community Dynamics by High-Throughput Sequencing (HTS) Metabarcoding during Flax Dew-Retting

**DOI:** 10.3389/fmicb.2017.02052

**Published:** 2017-10-20

**Authors:** Christophe Djemiel, Sébastien Grec, Simon Hawkins

**Affiliations:** Univ. Lille, Centre National de la Recherche Scientifique, UMR 8576 - Unité de Glycobiologie Structurale et Fonctionnelle, Lille, France

**Keywords:** flax dew-retting, bacterial and fungal microbiota dynamics, 16S rRNA and ITS amplicons, metabarcoding, high-throughput sequencing, HTS, CAZyme predictions, trophic modes

## Abstract

Flax dew-retting is a key step in the industrial extraction of fibers from flax stems and is dependent upon the production of a battery of hydrolytic enzymes produced by micro-organisms during this process. To explore the diversity and dynamics of bacterial and fungal communities involved in this process we applied a high-throughput sequencing (HTS) DNA metabarcoding approach (16S rRNA/ITS region, Illumina Miseq) on plant and soil samples obtained over a period of 7 weeks in July and August 2014. Twenty-three bacterial and six fungal phyla were identified in soil samples and 11 bacterial and four fungal phyla in plant samples. Dominant phyla were Proteobacteria, Bacteroidetes, Actinobacteria, and Firmicutes (bacteria) and Ascomycota, Basidiomycota, and Zygomycota (fungi) all of which have been previously associated with flax dew-retting except for Bacteroidetes and Basidiomycota that were identified for the first time. Rare phyla also identified for the first time in this process included Acidobacteria, CKC4, Chlorobi, Fibrobacteres, Gemmatimonadetes, Nitrospirae and TM6 (bacteria), and Chytridiomycota (fungi). No differences in microbial communities and colonization dynamics were observed between early and standard flax harvests. In contrast, the common agricultural practice of swath turning affects both bacterial and fungal community membership and structure in straw samples and may contribute to a more uniform retting. Prediction of community function using PICRUSt indicated the presence of a large collection of potential bacterial enzymes capable of hydrolyzing backbones and side-chains of cell wall polysaccharides. Assignment of functional guild (functional group) using FUNGuild software highlighted a change from parasitic to saprophytic trophic modes in fungi during retting. This work provides the first exhaustive description of the microbial communities involved in flax dew-retting and will provide a valuable benchmark in future studies aiming to evaluate the effects of other parameters (e.g., year-to year and site variability etc.) on this complex process.

## Background

Land plants fix ~123 billion tons of carbon per year (Beer et al., 2010) of which an important part becomes channeled into the production of lignocellulosic biomass in plant cell walls (Kuhad and Singh, 1993; Boerjan et al., 2003; Zhou et al., 2011). Soil microflora function as key decomposers in various ecosystems (Soliveres et al., 2016) and are able to degrade this biomass, consisting of lignin and polysaccharide polymers such as cellulose, hemicelluloses, and pectins, by producing a set of synergistically acting hydrolytic enzymes (Warren, 1996; Lynd et al., 2002; Kubicek et al., 2014; Cragg et al., 2015).

During this process, monosaccharides are released and used by microorganisms for energy production thereby contributing to maintenance of the carbon cycle. The microbial diversity associated with this biomass degradation can vary depending on plant cell wall structure and the stage of the decomposition (Akin, 2008; Ventorino et al., 2015; Montella et al., 2017). Microbial dynamics can also vary depending on site location, soil composition, plant species, and biomass architecture (Schneider et al., 2012; Voříšková and Baldrian, 2013; Cardenas et al., 2015; Ventorino et al., 2015). Several investigations have reported that fungal communities change during leaf (e.g., beech, oak, maize) litter decay with an initial predominance of species assigned to the Ascomycota phylum, replaced gradually by Basidyomycota (Schneider et al., 2012; Kuramae et al., 2013; Voříšková and Baldrian, 2013). Bacterial dynamics generally involve changes in the relative proportions of species assigned to Proteobacteria, Actinobacteria, and Bacteroidetes depending on sampling location and wood species to be degraded (Ventorino et al., 2015). The composition and succession of different microbial communities is presumably related to their capacity to degrade and utilize biomass present at a given moment. In this context it is interesting to note that particular microbial CAZymes such as endo- and exo-cellulases, xylanases, pectinases, and peroxidases have been associated with specific ecological groups during plant cell wall decomposition (Eastwood et al., 2011; Zhao et al., 2014; Ventorino et al., 2015).

In this study we investigate the microflora associated with a particularly interesting, and ancient example of human exploitation of microbial lignocellulose degradation known as retting that is believed to date back to the Upper Paleolithic and/or Neolithic (Gübitz and Cavaco-Paulo, 2001; Kvavadze et al., 2009). This process is still used today and constitutes the first step in the industrial separation of long bast fibers from the stems of different fiber species such as flax, hemp, jute, and kenaf (Md. Tahir et al., 2011) used for textiles and composites (Campilho, 2015; Pil et al., 2016). During retting, bast fiber bundles become progressively separated from the surrounding stem tissues, and inter-fiber cohesion is reduced via the action of hydrolytic enzymes produced by straw-colonizing microorganisms (Rosemberg, 1965; Zhang et al., 2005; Md. Tahir et al., 2011; Akin, 2013; Preisner et al., 2014). Retting is performed by either leaving plants on the soil (dew- or field-retting), or by placing them in ponds, rivers, or water tanks (water-retting). Although good quality fibers are produced by water retting this process is more labor intensive and associated with extensive water pollution. Currently the majority of the world's flax fiber is produced by dew-retting (Akin, 2013; Preisner et al., 2014).

The main challenge during retting is to facilitate fiber de-cohesion without degrading cellulosic fibers by over-retting (Brown and Sharma, 1984; Akin et al., 1998; Henriksson et al., 1999). Since this process relies on enzymes produced by colonizing microorganisms, a better knowledge of the different groups/species involved should enable a greater understanding and control of this complex process. Although various bacteria and fungi have been identified in a number of different studies by using isolation and culturing approaches (Sharma, 1986a; Henriksson et al., 1997), such a strategy is not powerful enough to obtain a complete inventory of the microorganisms present as only a small percentage of taxa can be successfully cultured under laboratory conditions (Staley and Konopka, 1985; Amann et al., 1995). More recently, molecular tools such as 16S rRNA gene amplification were used to identify new bacteria during bamboo, hemp, and flax retting (Tamburini et al., 2003; Fu et al., 2011; Ribeiro et al., 2015) and 18S rRNA gene amplification was used to identify fungi during hemp retting (Ribeiro et al., 2015). Nevertheless, these approaches are unable to generate an exhaustive inventory of the retting microbiome.

Over the last decade, microbial ecology studies have greatly benefited from the use of high throughput sequencing (HTS) technologies that can produce an exhaustive inventory of bacteria and fungi from complex samples such as soil, litter compost, rumen, and the midgut of cellulosic-feeding insects via targeted-metagenomics (Hirsch et al., 2010; Ihrmark et al., 2012; Suenaga, 2012). These approaches were also used to study plant-microbe interactions (Knief, 2014; Peršoh, 2015) in rhizospheres or endospheres (Lundberg et al., 2012; Bodenhausen et al., 2013; Beckers et al., 2017). Only two studies have reported the use of HTS technologies (Ion Torrent PGM system) on kenaf retting (Visi et al., 2013) and more recently during water-retting of flax (Zhao et al., 2016). In both studies, the microbial community analysis was limited to bacterial domain, despite the importance of fungal taxa in the production of extracellular hydrolytic enzymes (Schneider et al., 2012). In this work, we report the first exhaustive HTS microbial inventory focusing on both bacterial and fungal communities using rRNA amplicon sequencing during dew-retting of flax.

## Methods

### Experimental design–study site–sampling

Flax plants (*Linum usitatissimum* L., Cultivar Lorea) were sown on 14 March 2014 near Martainneville (F-27210 Region Hauts-de-France) in the north of France (50°00′03″N and 1°42′27″E). Plants were cultivated and retted on a typical silt loam soil with a neutral/slightly acid pH (INRA Soil Analysis Laboratory, LAS, Arras, France, http://www.lille.inra.fr/las) (Supplementary Table 1). Climatic data during the retting period was obtained from the Abbeville meteorological station at 10 km from Martainneville (infoclimat: http://www.infoclimat.fr/observations-meteo/temps-reel/abbeville/07005.html) (Supplementary Figure 1). “Early” and “standard” flax cultures were pulled (up-rooted) on the 16.07.2014 and 24.07.2014, respectively and dew-retted in the field until the 25.08.2014 (early cultures) and 05.09.2014 (standard cultures). Replicate straw (plant) and soil samples were collected at regular intervals (R0–R6) during retting from five different locations in the retting field chosen according to a non-systematic W pattern as previously described (Plassart et al., 2012) and shown in Supplementary Figure 2. For straw samples, the middle region (30 cm long × total swath height) of the swath was collected; for soil samples, cores (20 cm deep × 8 cm diameter) were used. Stem samples were directly stored at −20°C and soil samples were sieved (pore size <2.0 mm), homogenized and freeze-dried before storage at −80°C.

### DNA extraction

DNA was extracted from 1 g sample using the GnS-GII (Plassart et al., 2012; Terrat et al., 2012, 2015). Briefly, samples were ground in 15 ml Falcon tubes containing a bead mix (ceramic, silica, and glass) and lysis buffer (100 mM Tris-HCl, pH 8; 100 mM EDTA, pH 8; 100 mM NaCl, 2% w/v and sodium dodecyl sulfate, 2% w/v) in a FastPrep®-24 (MP-Biomedicals, NY, USA) (3 × 30 s at 4, 000 s^−1^ shaking). Proteins were precipitated by adding 100 μl of KAc (3 M) and nucleic acids recovered by isopropanol precipitation and washed with 70% ethanol, before drying and re-suspension in 100 μl water.

### DNA purification, quantification, and normalization

DNA extracts were filtered through PVPP (PolyVinylPolyPyrrolidone) Micro Bio-Spin® Columns with Bio-Gel® P-6 (Bio-Rad) by a 4 min at 1,000 g, 10°C centrifugation. Collected samples were then purified using the Geneclean Turbo kit (MP-Biomedicals, NY, USA) following the manufacturer's instructions. DNA was quantified on a LightCycler 480 System (Roche) using the Quant-iT™ PicoGreen® dsDNA Assay kit (Invitrogen). Samples were normalized to a concentration of 5 ng/μl and the DNA from the five replicates pooled using the epMotion® 5075 TMX (eppendorf). Altogether, 16 soil samples and 14 stem samples were recovered for further analysis.

### Primers, PCR amplification, and sequencing

Bacterial 16S rDNA were amplified using the forward primer S-D-Bact-0341-a-S-17 described by Klindworth (Klindworth et al., 2013) coupled with a customized reverse primer S-D-Bact-0787-a-A-19, based on the 786r primer (Gołebiewski et al., 2014). Fungal ITS regions were amplified using the fITS7 forward primer 5.8S (Ihrmark et al., 2012) and the reverse primer ITS4_KYO1 (Toju et al., 2012; Bokulich and Mills, 2013). All primer sequences are given in Supplementary Table 2.

Amplifications were carried out in a total volume of 40 μl using 5 ng of DNA, 4 μl of 5x HOT FIREPol ® Blend Master Mix with 7.5 mM MgCl_2_ (Solis Biodyne, Tartu, Estonia), 0.8 μl (0.2 μM) of each primer. PCR1 conditions were: 15 min at 95°C, followed by 30 cycles of 20 s at 95°C, 30 s at 53°C, and 20 s at 72°C, and final elongation for 5 min at 72°C. Single multiplexing was performed using home-made 6 bp indexes that were added to reverse primer during a second PCR2 of 12 cycles using indexed primers. The resulting PCR2 products were purified by HighPrep™ PCR (Magbio) clean-up system as described by the manufacturer, pooled and loaded onto the Illumina MiSeq cartridge according to the manufacturer instructions for a 2 × 250 bp paired-end sequencing on the GeT-PlaGe Genotoul Platform (INRA Castanet Tolosan, France). The quality of the run was checked internally using PhiX, and then each pair-end sequence was assigned to its sample with the help of the previously integrated index.

### Sequence processing

A bioinformatic pipeline based on mothur v.1.37.4 (https://github.com/mothur/mothur/releases) (Schloss et al., 2009) was configured to process the bacterial 16S rRNA gene sequences. This pipeline uses the standard Schloss lab operating procedure (http://www.mothur.org/wiki/MiSeq_SOP). Pair-End (PE) FASTQ files were overlapped to form contiguous reads in a single FASTA file with zero differences to the primer sequence and a quality score threshold of 30. Sequences with the following characteristics were removed: ambiguous bases and mismatches, <300/> 500 bp, homopolymers >8 bp, overlap <30 bp. Bacterial sequences were aligned against both SILVA (SSU SILVA 123) and Greengenes (August 2013 release, for input PICRUSt) reference databases. A pre-clustering was done to reduce noise as recommended (Pruesse et al., 2007; Huse et al., 2010) allowing for up to four differences between sequences. Chimeras were detected and removed *de novo* with the UCHIME (version 4.2) algorithm (Edgar et al., 2011). The clustering of the non-chimeric sequences to Operational Taxonomic Units (OTUs) was done by *de novo* clustering at 0.03 cut-off of dissimilarity using neighbor based on genomic distance matrix. Finally, a general count sequence table for each OTU of all samples was generated to obtain the consensus taxonomy based on the Ribosomal Database Project's naïve bayesian classifier method (Wang et al., 2007) and for the future OTU-based analysis.

For processing ITS2 from fungal ribosomal ITS sequences, the recently described PIPITS v.1.3.3 pipeline (https://github.com/hsgweon/pipits/releases) was used (Gweon et al., 2015). Raw reads were prepared for ITS extraction and the chosen sub-regions extracted with the ITSx software tool (Bengtsson-Palme et al., 2013) before clustering and taxonomic assignation using the UNITE database (version 31.01.2016) (Abarenkov et al., 2010).

All parameters, algorithms and tools for the bioinformatic steps used in the two pipelines are given in Supplementary Table 3.

The microbial DNA sequencing data sets supporting the results in this article are available at the EBI ENA with accession number PRJEB20299.

### Statistical analysis

All estimators used to measure the α-diversity and β-diversity were calculated applying mothur procedures following recommendations and parameters suggested by tutorials (Kozich et al., 2013).

Alpha-diversity was estimated with the chao1 non-parametric estimator (Chao, 1984) and evenness was measured with Heip's estimator [E_heip_ = (e^H′^ − 1)/(S-1) with H' being Shannon's diversity index and S the number of species] (Heip, 1974). Community diversity was estimated with Shannon's diversity index (Ludwig and Reynolds, 1988) and the inverse Simpson's index (Simpson, 1949). Microbial community coverage was tested by calculating the Good's non-parametric coverage estimator (Good, 1953; Esty, 1986) and verified by rarefaction curves. Differences in alpha diversities were evaluated using the Mann-Whitney-Wilcoxon test.

Beta-diversity was assessed using the Yue and Clayton theta similarity coefficient for community structure and the Jaccard index for community membership (Yue and Clayton, 2005; Barwell et al., 2015).

The non-parametric analysis of molecular variance (AMOVA) (Excoffier et al., 1992) was used to examine the significance of differences between and within different groups (Early vs. Standard and Before vs. After turning swaths) with a *p*-value ≤0.05 being considered as statistically significant.

The diversity indices are computed from a standardized file containing the count of OTUs for each sample.

Spearman rank correlation coefficients were calculated from generated dissimilarity matrices to look for any significant correlations between climatic conditions (temperature and rainfall) and bacterial and fungal community structure.

For population level analyses, several tools were used: Metastats (White et al., 2009) (White et al., 2009), LEfSe (Linear discriminant analysis Effect Size (Segata et al., 2011), and Indicator (from Mothur software).

The PICRUSt v.1.0.0 (https://github.com/picrust/picrust/releases) pipeline (http://picrust.github.io/picrust/) (Langille et al., 2013) was used to predict the functional composition of bacterial enzymatic activity abundance using 16S rDNA datasets. An OTUs table (input file) in BIOM format was generated using Mothur and then reference picked against the Greengenes database. Accuracy of metagenome predictions was controlled by measuring the weighted Nearest Sequenced Taxon Index (NSTI) scores that reflect the availability of reference genomes closely related to the most abundant microorganisms for each sample. To analyze the Carbohydrate Active enZymes (CAZymes) prediction, a pre-calculated table was used (https://sourceforge.net/projects/picrust/files/precalculated_files/).

The FUNGuild v1.0 database (https://github.com/UMNFuN/FUNGuild) was used to assign ecological functions (trophic modes) to each OTUs (Nguyen et al., 2016).

Graphic representations were produced using handmade scripts and based on Highcharts facilities (http://www.highcharts.com/) and jvenn plug-in (Bardou et al., 2014).

## Results

### Metabarcoding and sequencing

16S rDNA (bacterial) and ribosomal ITS (fungal) amplicons were sequenced using the Illumina MiSeq system. Redesigned primers (Supplementary Table 2) were used in order to avoid potential amplification of plant chloroplastic/mitochondrial DNA. Sequencing generated a very large data set ranging between 103,859 and 279,553 (average = 162,390 ± 33,164) bacterial raw sequences and between 187,055 and 483,703 (average = 285,070 ± 62,420) fungal raw sequences (Supplementary Tables 4A,B). OTU tables listing all OTUs detected and their abundance normalized by a subsampling are given in Supplementary Table 5 (bacteria) and Supplementary Table 6 (fungi).

### Community coverage and diversity

To estimate how representative our samples were of the bacterial and fungal communities Good's coverage estimator was calculated for all samples (Supplementary Tables 6, 7). For bacterial samples Good's coverage values were greater than 99% for all straw samples and between 91 and 92% for soil samples indicating: (i) the high coverage of the sampling community and (ii) that the redesigned reverse primer did not significantly affect V3-V4 bacterial amplification (Supplementary Table 6). For fungal samples, Good's coverage estimators were above 99% for all samples confirming that the population is well-sampled (Supplementary Table 7). These results indicate that the sequencing depth used provides an accurate view of microbial community diversity and were also confirmed by the rarefaction curves (Supplementary Figures 3,4).

To analyze community diversity (alpha diversity) within our microbial samples we calculated Chao1 (species richness), Heip's (species evenness), and Inverse Simpson index metric estimators (Supplementary Figures 5, Supplementary Tables 7–9). For all estimators and all conditions [soil vs. plant (straw), early vs. standard cultures] community diversity was always higher in bacterial samples when compared to fungal samples. For both bacterial and fungal samples all indicators indicated that community diversity was higher in soil samples when compared to plant samples. In contrast, the same indicators revealed no difference in bacterial community diversity (both soil and plant samples) between early vs. standard cultures. For fungal communities, the situation was more complex. While no differences in species richness (Chao1) were observed between early vs. standard plant samples, Heip's estimator values suggested differences in sample evenness. The Inverse Simpson index values also suggested differences in community diversity between these two samples.

Examination of indicator values during the retting period (R0–R6) revealed a range of different profiles suggesting that sample community diversity evolves during this process (Supplementary Figure 5). When only the Inverse Simpson index is taken into account as an overall measure of community diversity (Supplementary Tables 7, 8) all profile types show an overall bimodal form with a peak/trough mainly occurring at R2 (5/8 profiles), but also at R3 (2/8 profiles) and R1 (1/8 profiles). Taken together, these results would suggest that sample community diversity changes (increases or decreases) at some point after R2 (and/or R3). The R2/R3 points are close to the moment when the stem swathes were turned and the observed change in community diversity values might be related to this process. When “early” and “standard” culture sample values are pooled (to provide sufficient data points) analyses shows that there is a significant effect (*p*-value <0.05) of swath turning on bacterial, but not fungal community diversity.

Comparison of soil Chao1, Heip's and Inverse Simpson estimators for the first retting point (R0) with those obtained at the sowing stage (R-1) (Supplementary Figure 5) show that both bacterial and fungal community diversity are always lower at R-1. Such an observation suggests that flax plants modify soil diversity through either a rhizosphere effect and/or the input of other organic material (e.g., leaves). Nevertheless, other abiotic effects (e.g., temperature, soil moisture content) may also have an effect and should not be neglected.

### Community membership and structure

**Figure 1:**
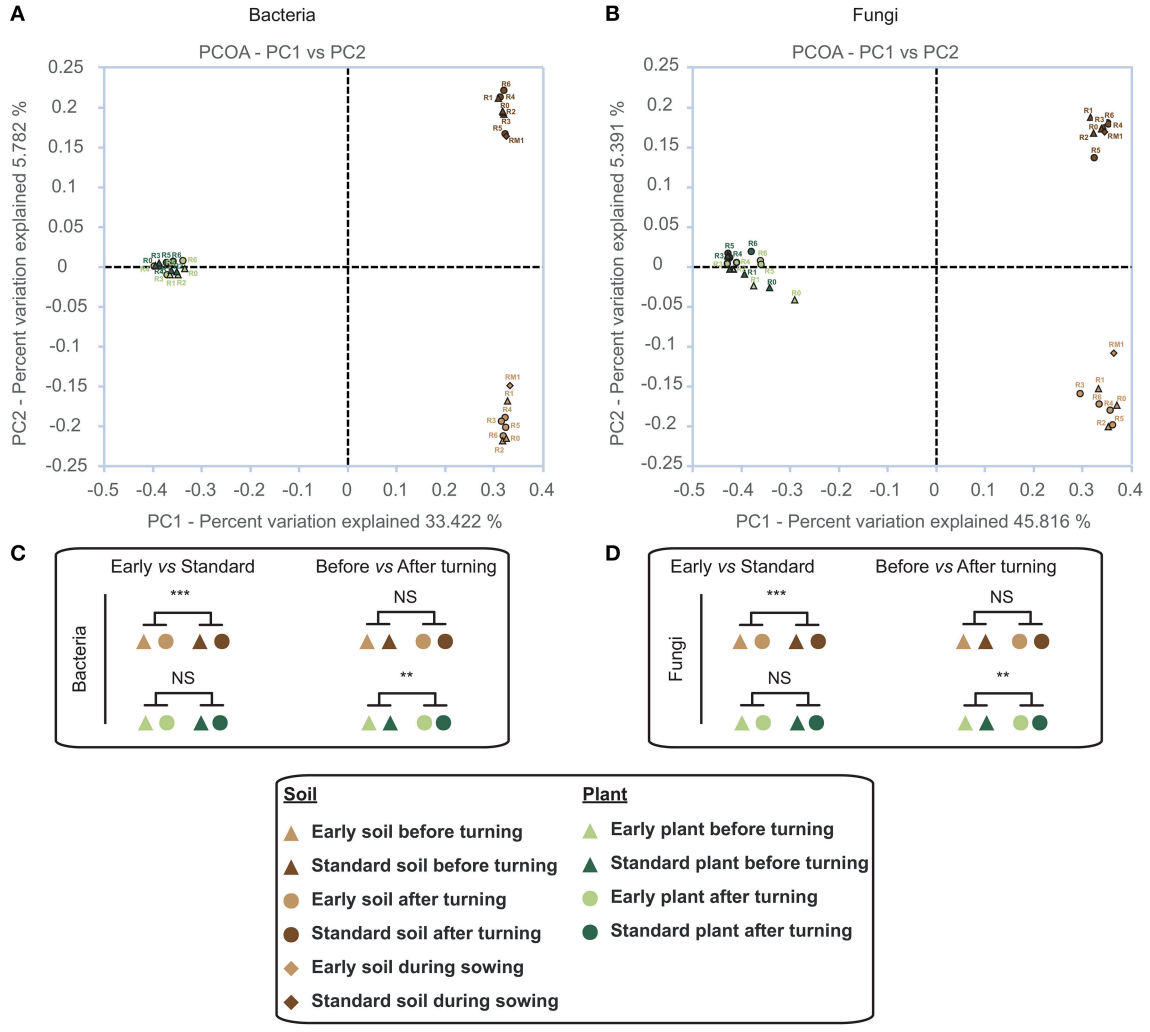
Principal coordinate analysis (PCoA) of bacterial **(A)** and fungal **(B)** community membership based on Jaccard distances. Different colors indicate the source samples (light brown, soil early harvest; dark brown, soil standard harvest; light green, plant early harvest; dark green, plant standard harvest). Triangles, samples before swath turning; circles, samples after swath turning; lozenge, soil sample during sowing. **(C,D)** statistically significant clustering based on AMOVA.

To obtain an idea of the beta diversity between our samples we analyzed bacterial/fungal community membership and structure. Principal Coordinate analysis (PCoA) using Jaccard distances (Figure 1) clearly revealed that membership between soil samples and plant samples differed for both bacterial (Figure 1A) and fungal (Figure 1B) communities. While no differences in community membership could be observed between early vs. standard culture plant samples (AMOVA centroid with *p*-value ≥ 0.05) the presence of two distinct clusters indicated that community membership clearly differed between early vs. standard soil samples. Statistical analyses also indicated that swath turning had a significant effect (AMOVA centroid with *p*-value < 0.05) on both bacterial and fungal community membership of plant samples, but not soil samples.

**Figure 2:**
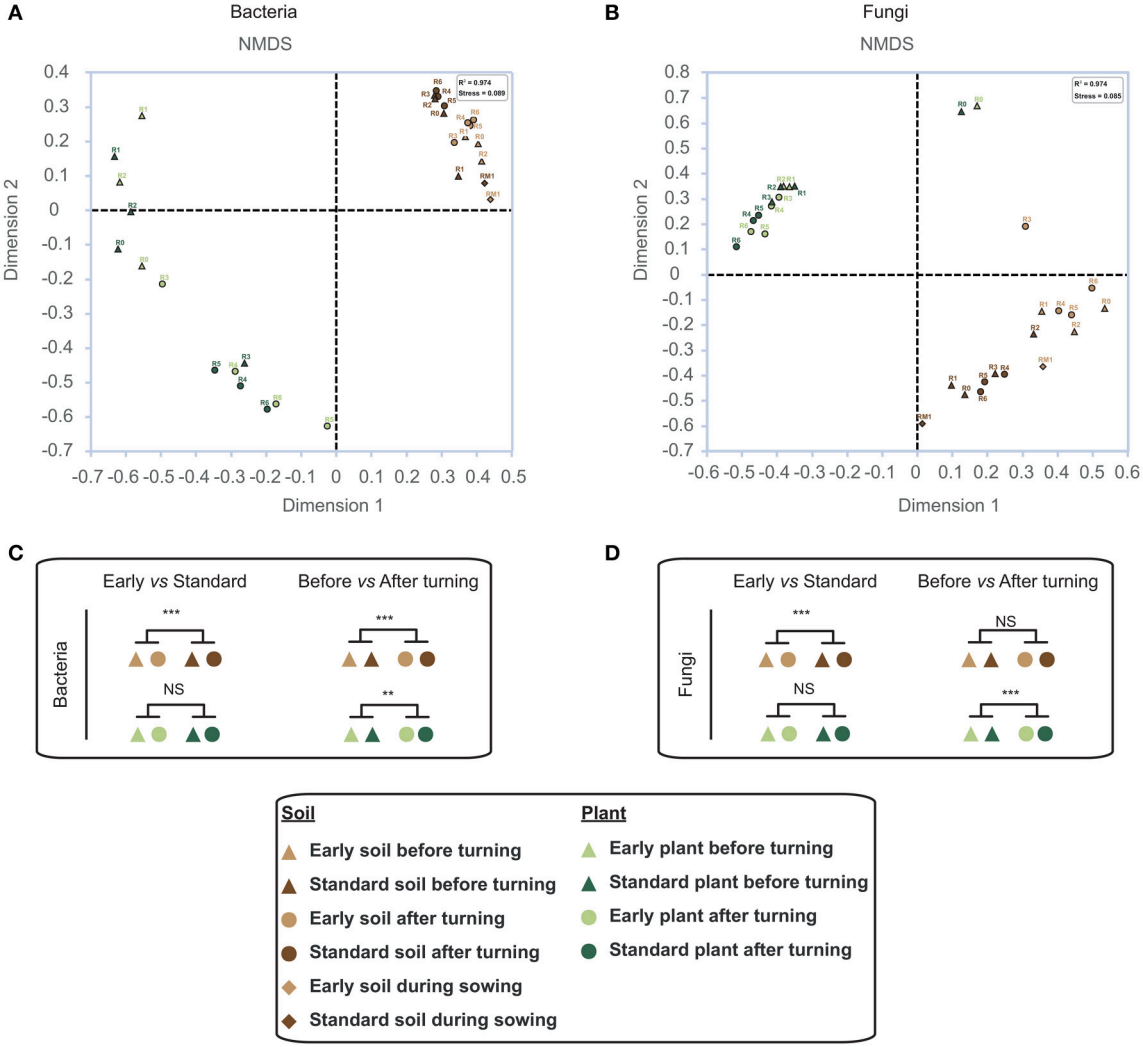
Non-metric multidimensional scaling (NMDS) of bacterial **(A)** and fungal **(B)** community structure based on Yue & Clayton distance matrix. Different colors indicate the source samples (light brown, soil early harvest; dark brown, soil standard harvest; light green, plant early harvest; dark green, plant standard harvest). Triangles, samples before swath turning; circles, samples after swath turning; lozenge, soil sample during sowing. **(C,D)** statistically significant clustering based on AMOVA.

To analyze community structure we then used the Non-parametric MultiDimensional Scaling (NMDS) ordination of Yue & Clayton dissimilarities to determine distance matrices (Theta YC distances) between all samples (Figure 2). The results show that stress values for both bacterial (0.089) and fungal (0.085) communities are inferior to 0.1 as recommended by Mothur SOP (Standard Operating Procedure, https://www.mothur.org). Overall, and as observed for community membership data, clear differences in community *structure* occur between soil and plant samples (early and standard cultures) for both bacterial (Figure 2A) and fungal (Figure 2B) samples. However, the fungal R0 (early and standard cultures) plant samples form a separate cluster from the other plant samples whereas bacterial R0 plant samples do not. The community structure of early and standard bacterial/fungal soil samples, but not plant samples, is also significantly different (*p*-value < 0.001). As observed for community membership, swath turning also appeared to modify community structure, but not necessarily in the same samples. For bacteria, swath turning had a significant effect on community structure in both soil and plant samples (cf. community membership, significant effect only in plant, but not soil, samples). In contrast for fungi, swath turning only had a significant effect on the community structure of plant, but not soil samples. Calculation of Spearman rank correlation coefficients indicated that there was no significant correlation between climatic conditions (temperature, rainfall) and community structure (Supplementary Table 10).

### Taxonomic distribution of identified bacteria and fungi

**Figure 3:**
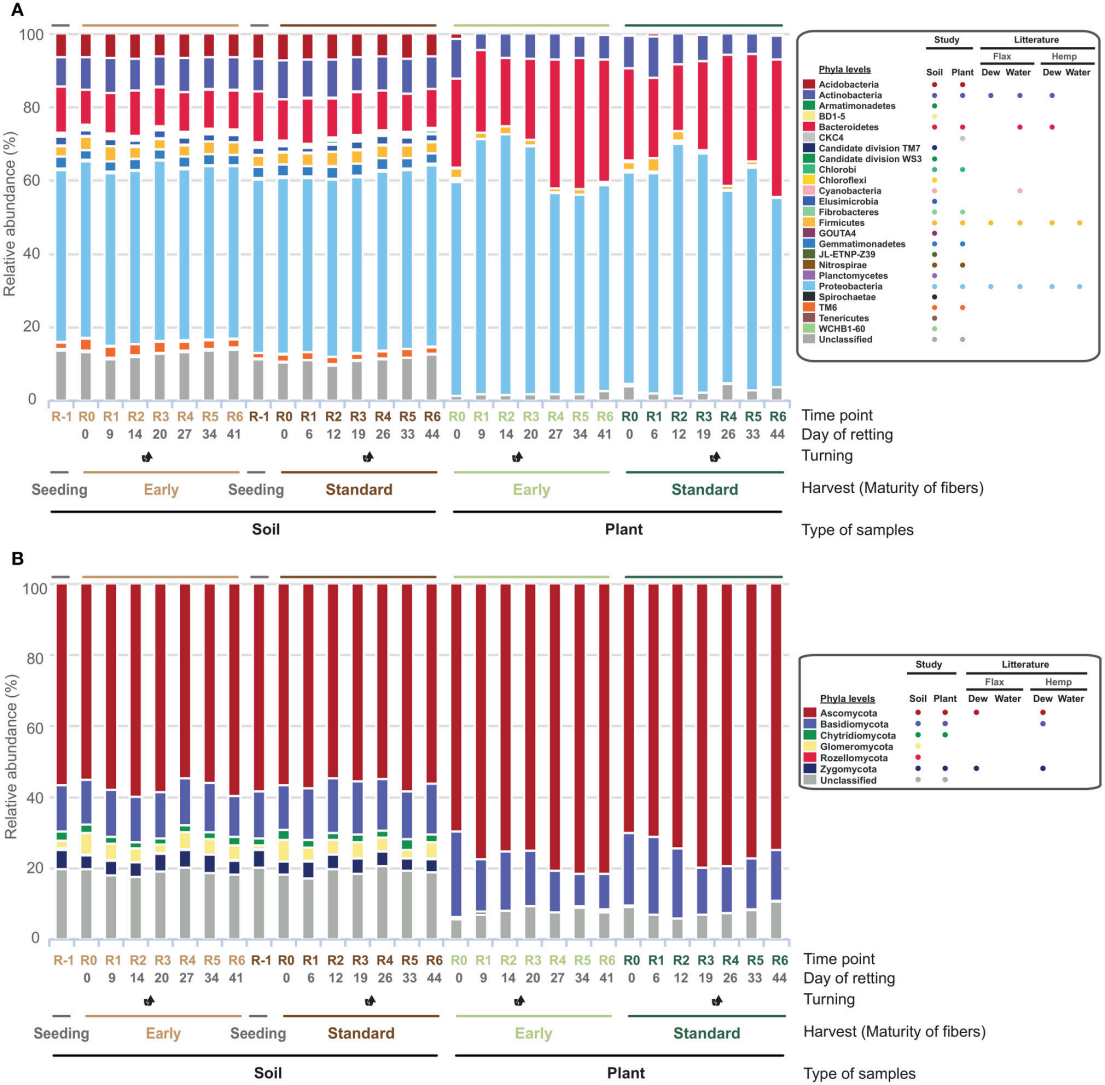
Bacterial **(A)** and fungal **(B)** relative abundance of OTUs at the phyla level (soil samples *n* = 16, and plant samples *n* = 14). The consensus taxonomy for bacterial OTUs was assigned from the SILVA database and for fungal OTUs from the UNITE database.

To evaluate taxonomic distribution of identified bacteria and fungi, OTUs were analyzed to determine consensus taxonomy (Figure 3). Overall more phyla (bacteria and fungi) were present in soil samples when compared to plant samples with 23 (excluding unclassified) bacterial and six fungal phyla in soil samples and 11 bacterial and four fungal phyla in plant samples. Of these phyla, 8 (bacteria) and 2 (fungi) were not previously associated with flax dew-retting in the literature thereby underlining the interest of a metabarcoding approach for the identification of new microorganisms. Although the number of phyla identified in soil samples was higher than in plant samples, the most abundant taxa were the same in both cases as might be expected in an analysis at this level (Phyla): Bacteria—Proteobacteria (x 60.64% ± 6.64); Fungi—Ascomycota (x 76.29% ± 4.047) (Supplementary Tables 11, 12). For both bacteria and fungi, the type of culture (early vs. standard) appeared to have little effect on phyla relative abundance, neither in soil nor in plant samples. The relative abundance in both bacterial and fungal soil samples appeared to remain fairly constant throughout the retting period. In contrast, relative abundance in bacterial *plant* samples was more dynamic being characterized by a relative increase and/or decrease in percentage relative abundances of Proteobacteria and Bacteroidetes at R2 (Figure 3A). The relative abundance in fungal plant samples appeared to be more stable throughout retting.

**Figure 4:**
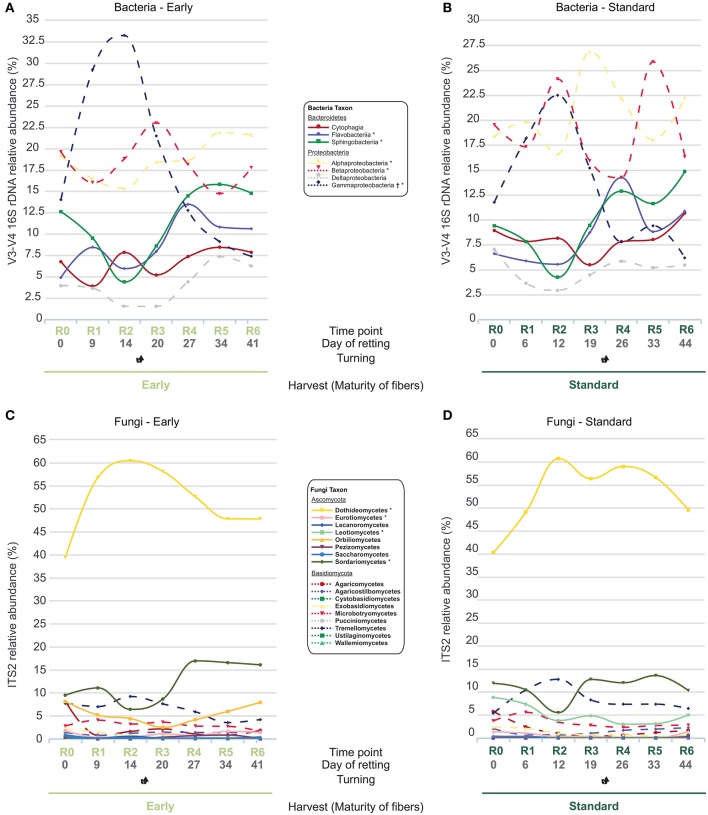
Dynamics of V3-V4 16S rDNA and ITS2 gene relative abundance of different classes in the main bacterial **(A,B)** and fungal phyla **(C,D)** in straw samples. **(A,C)** early harvest, **(B,D)** standard harvest. Classes previously associated with flax dew-retting^†^and water-retting^*^.

Subsequent analyses of plant samples at class level (Figure 4) indicated that the observed increase (Figure 3) in the % relative abundance of the Proteobacteria at R2 was mainly related to a substantial increase (>100%) in the relative abundance of the Gammaproteobacteria class in early samples (Figure 4A) correlated with a smaller reduction in relative abundances of the Flavobacteria and Sphingobacteria classes. Similarly, in standard samples the previously observed increase in Proteobacteria (Figure 4) could be related to the increase in Gammaproteobacteria and Betaproteobacteria, coupled with a decrease in relative abundance of Sphingobacteria at R2 (Figure 4B). Additional Proteobacteria peaks were also observed at R3 (Alphaproteobacteria) and R5 (Betaproteobacteria) but had less overall impact on the Proteobacteria/Bacteroidetes ratio in standard cultures due to an increase in relative abundance of Bacteroidetes classes in latter stages of retting.

Examination of relative abundances of fungal classes revealed a different pattern. Although more classes were identified (8/9 classes in the Ascomycota and Basidiomycota, respectively), the class Dothidiomycetes was by far the dominant class in both early (Figure 4C) and standard (Figure 4D) samples with a relative abundance ranging from a “low” of 40% (R0) and arriving at a maximum of 60+ % (R2). The Sordariomycetes were the next most abundant class with a value of between 10 and 15% relative abundance.

**Figure 5:**
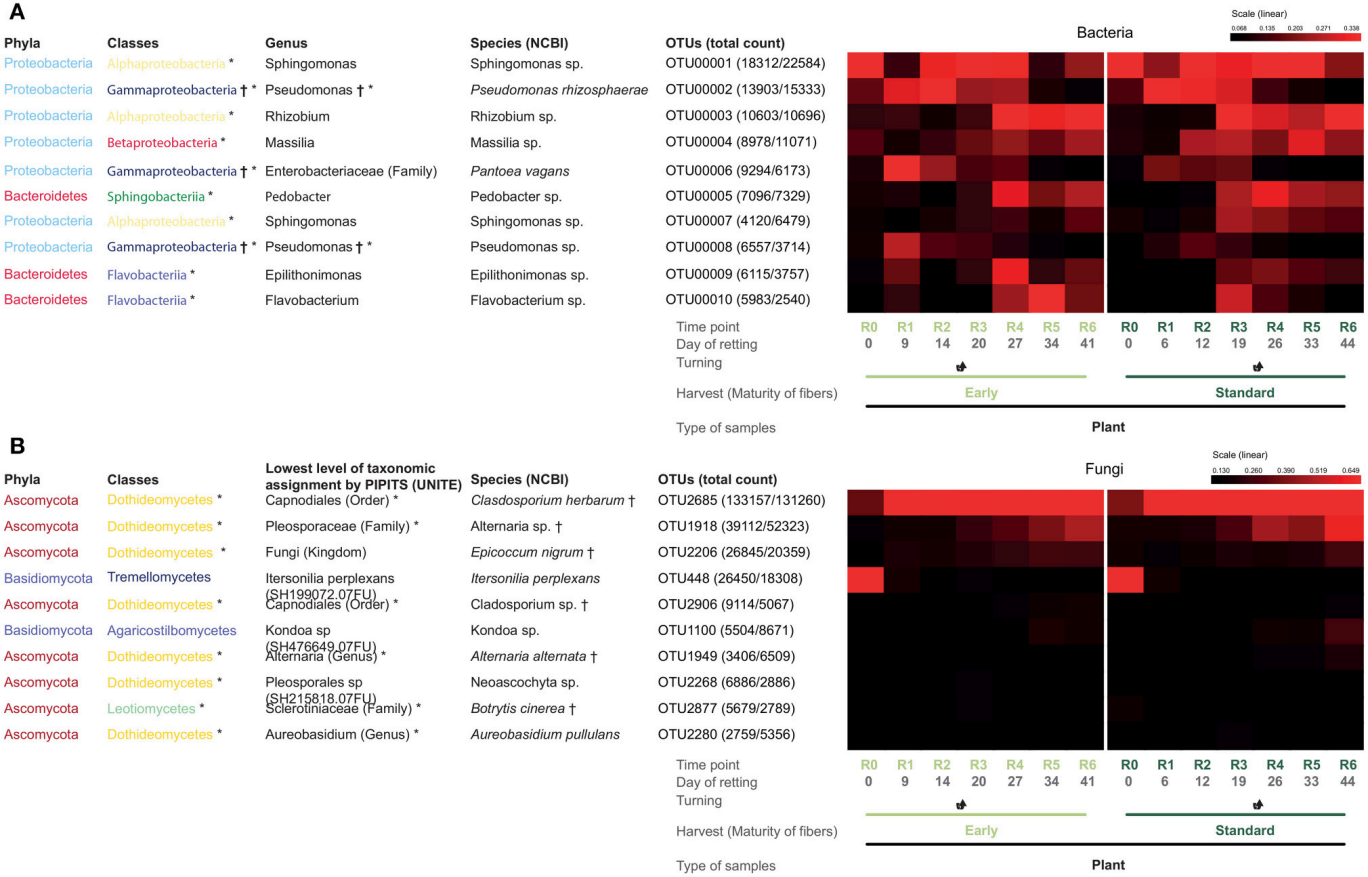
Heatmaps of the relative abundance of the top 10 OTUs of bacteria **(A)** and fungi **(B)** in plant samples (*n* = 14). OTUs previously associated with flax dew-retting^†^ and water-retting^*^.

The generation of community distance heatmaps (Figure 5) for the top ten bacterial and fungal OTUs provided more detailed information on the different taxonomic groups represented in classes identified in plant samples. For bacteria (Figure 5A), results underlined the abundance of Sphingomonas and Pseudomonas genera. For fungi (Figure 5B), the order Capnodiales represented by *Cladosporium herbarum* was clearly the most abundant group. When analyzed globally, three different profiles could be identified: (i) the OTU is present throughout the retting period (e.g., OTU00001, Sphingomonas sp. and OTU2685, *C. herbarum*); (ii) the OTU is present at the beginning and then decreases (e.g., OTU00002, *Pseudomonas rhizosphaerae* and OTU00006, *Pantoea vagans*); and (iii) the OTU is absent at the beginning and then increases during retting (e.g., OTU00003, Rhizobium genus, OTU00004, Massilia sp. and OTU1918, Altenaria sp.).

### Community-level analysis

Our results suggested that swath turning during retting had a significant effect on the microbial communities. To identify those OTUs most likely to explain differences highlighted by the diversity analyses between before- and after-turning - and that could therefore represent potential biomarkers of this process, we used three different tests (Metastas, LEfSE, and Indicator). Our results (Supplementary Figure 6, Supplementary Tables 13–15) identified 7/8 bacterial and fungal OTUs, respectively “before,” and 14/6 bacterial and fungal OTUs “after,” swath turning in all three tests. Four and six of these OTUs are present in the top 10 bacterial/fungal OTUs, respectively (Figure 5).

### Hydrolytic enzyme potential and trophic mode prediction

**Figure 6:**
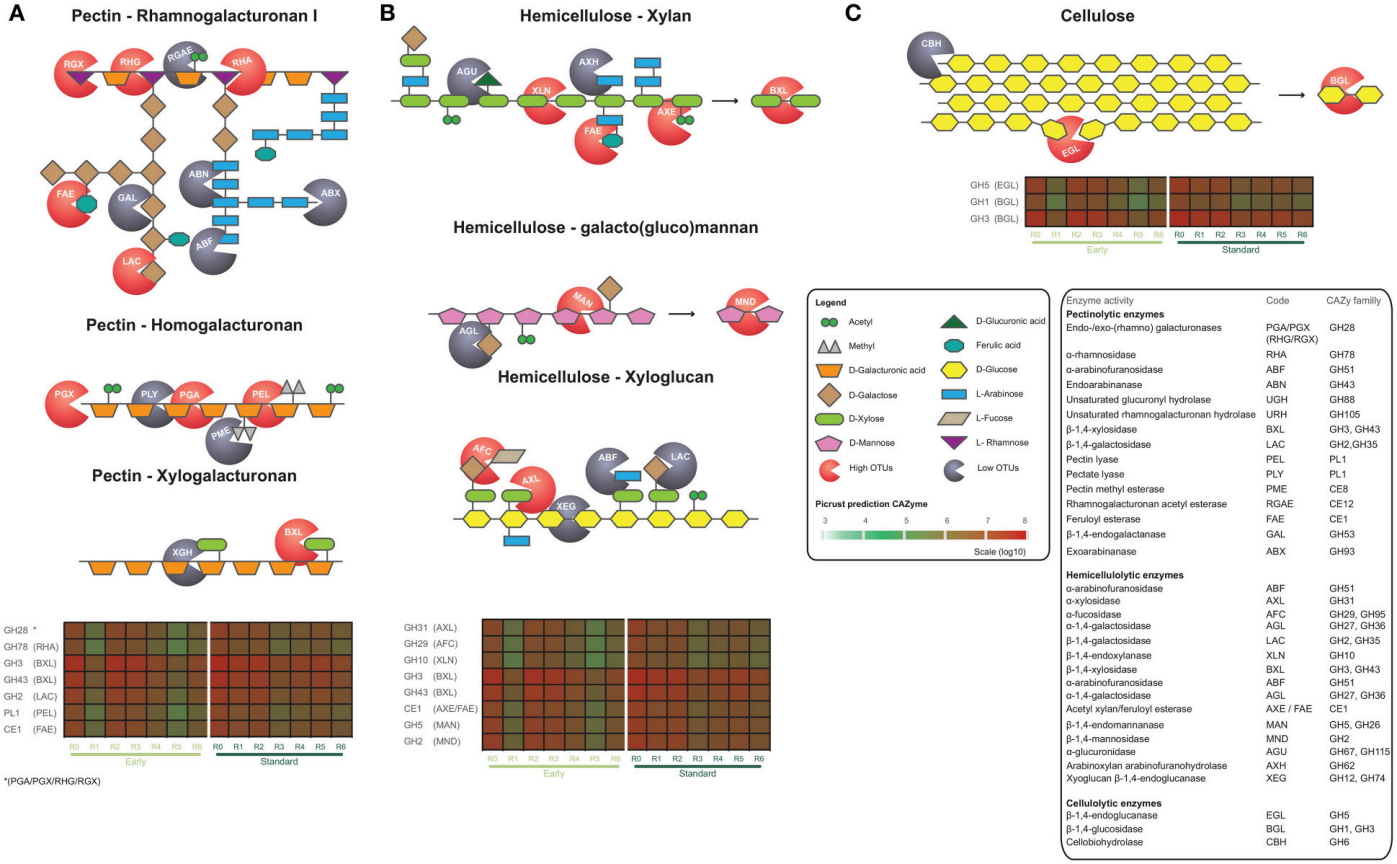
Schematic representation of plant cell wall polysaccharides and predicted bacterial hydrolytic enzymes present in flax straw samples. **(A)** pectins, **(B)** hemicelluloses, and **(C)** cellulose. Heatmaps show the total counts of the most abundant CAZyme families in early and standard harvests obtained from the OTUs table (Greengenes Database used for consensus taxonomy) and generated by PICRUSt software. Red “pacman”, high OTUs abundance enzymes; gray “pacman”, low OTUs abundance enzymes.

**Figure 7:**
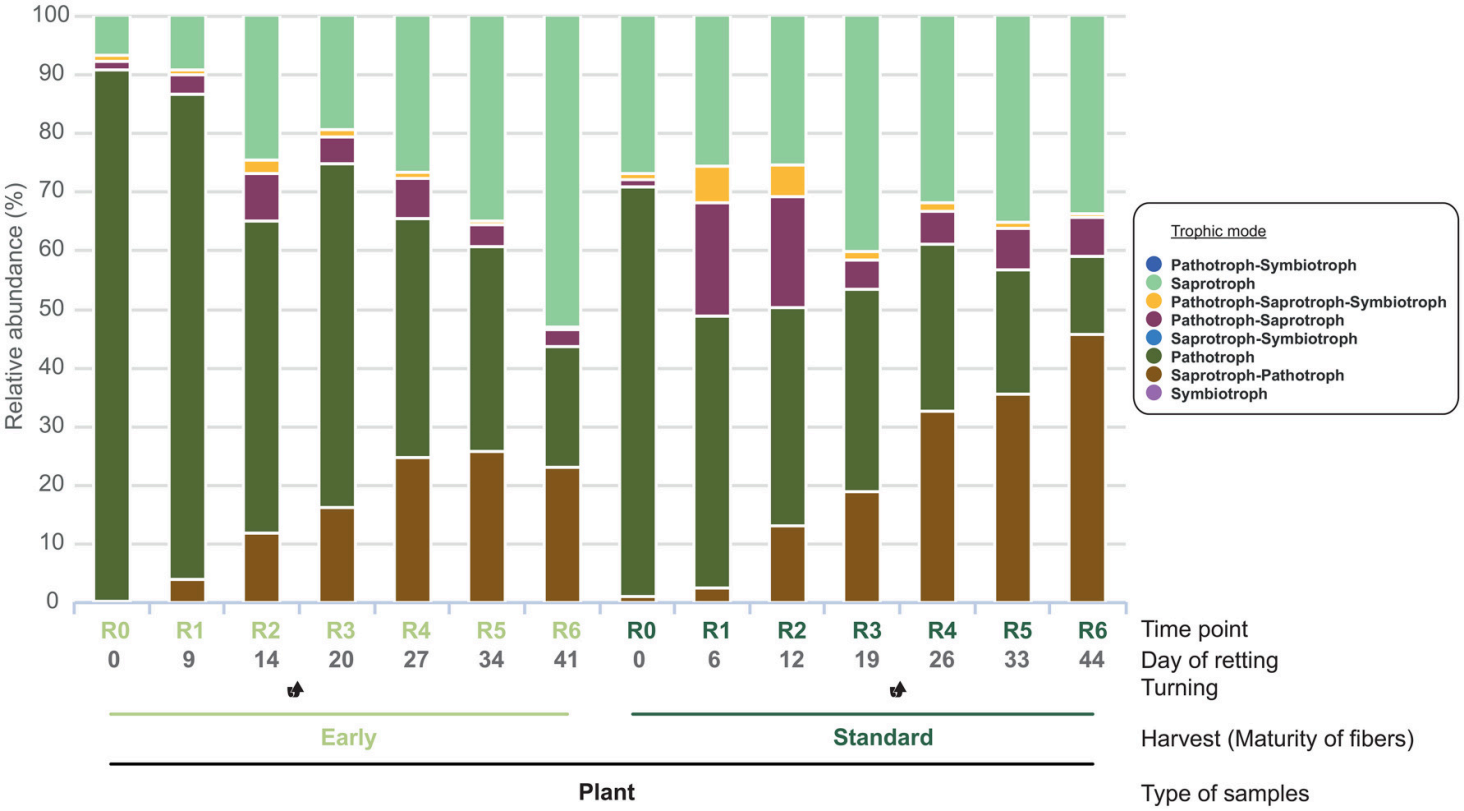
Relative abundance of fungal OTUs classified by trophic mode. The trophic mode was assigned using the FUNGuild database and based on 35% of OTUs (i.e., total = 1048).

Dew-retting of flax straw occurs via the action of hydrolytic enzymes produced by microorganisms and we therefore used PICRUSt software followed by expert curation to predict the bacterial Carbohydrate Active enZyme (CAZy) families potentially present during dew-retting and playing a role in the degradation of cell wall polymers. For all stem samples the NSTI scores were around the 0.15 level considered as acceptable according to PICRUSt instructions. Our results (Figure 6, Supplementary Figure 7) show that a wide range of different enzymes targeting both the backbones and side chains of the major polysaccharide cell wall polymers (cellulose, hemicelluloses, pectins) are present. Altogether, 22, 32, and 6 CAZy families targeting pectin, hemicelluloses and cellulose polymers were identified (Supplementary Figure 7). Generally, the hydrolytic enzyme potential (all polymers) was greater during the first stages of retting (R0-R2/R3) compared to latter stages (R3/R4–R6) for both “early” and “standard” cultures. The drop in hydrolytic potential observed for R1 and R5 stages in “early” cultures is most likely related to the corresponding decrease in the most abundant bacterial OTU (e.g., Sphingomonas OTU00001, Figure 5). PICRUSt prediction does not exist for fungal OTUs and so hydrolytic enzyme potential cannot be directly predicted. Nevertheless, we were able to gain a relative idea of the overall hydrolytic enzyme potential by using the FUNGuild software that describes fungal trophic mode. Our results (Figure 7) show a progressive decrease in relative abundance of pathotrophs associated with a steady increase in saprotrophs and saprotrophs-pathotrophs as retting progresses. Pathogenic fungi generally produce a wider range of cell wall degrading enzymes than rot fungi and observed change in trophic mode during retting could suggest a decrease in hydrolytic enzyme diversity (Choi et al., 2013).

## Discussion

### Microbial identification and retting parameters

Previous studies using culture-based approaches and non-HTS metabarcoding have identified different bacteria and fungi phyla present during retting including Actinobacteria, Firmicutes, Proteobacteria (bacteria), and Ascomycota and Zygomycota (fungi) (Lanigan, 1950; Rosemberg, 1965; Brown, 1984; Sharma, 1986a,b; Donaghy et al., 1990; Henriksson et al., 1997). Our results obtained using metabarcoding coupled with HTS not only identified these phyla, but also allowed the identification of new phyla not previously associated with dew-retting. Overall we identified 95 bacteria and 215 fungi species in dew-retted flax straw (plant) samples. HTS metabarcoding has been recently used to investigate bacterial (but not fungal) population dynamics in water-retted flax (Zhao et al., 2016). A comparison of relative abundances of the major bacterial phyla identified indicates that water retting is very different from dew-retting, despite the fact that the same lignocellulosic material is being degraded. Major phyla identified during water-retting were Firmicutes (genus Clostridium) and Proteobacteria (genera Azotobacter and Enterobacter). In contrast, Firmicutes were only present in low abundance during dew-retting and Azotobacter were absent. These differences can be most likely related to the anaerobic environment of water-retting compared to the more aerobic environment of dew-retting. Indeed, Clostridium is an obligate anaerobe and is known to be an agent of water-retting (Donaghy et al., 1990; Tamburini et al., 2003).

Phyla, identified in our study and not previously associated with flax dew-retting, included, for the bacteria, Acidobacteria, Bacteroidetes, CKC4, Chlorobi, Fibrobacteres, Gemmatimonadetes, Nitrospirae and TM6; and for the fungi, Basidiomycota and Chytridiomycota. The Bacteroidetes phylum has been associated with cellulose degradation in agricultural soils (Schellenberger et al., 2010) and was previously detected in hemp dew-retting (Ribeiro et al., 2015) and flax water-retting (Zhao et al., 2016). Our observation of this phylum could indicate that it is also involved in flax dew-retting. Basidiomycota are linked to plant cell wall degradation in different ecosystems (Baldrian et al., 2008; Schneider et al., 2012; Kuramae et al., 2013; Voříšková and Baldrian, 2013; Rytioja et al., 2014) and were also detected in hemp dew-retting (Ribeiro et al., 2015).

Although the observation that new bacterial phyla (except for the Bacteroidetes) and fungal phyla represent less than 2% of the whole microbiota might suggest that they are not involved in the retting process, some of these phyla are related to microorganisms characterized as biomass degraders in previous studies (Zhao et al., 2014). This observation, together with the fact that low abundance OTUs can still contribute to the decomposition of plant matter (Baldrian et al., 2012) indicates that these phyla should not be ignored during the study of dew-retting.

A number of parameters potentially affecting microbial population structure during retting were examined. It is commonly admitted by farmers that the maturity of flax plants has a direct impact on the retting time and influences the choice for the pulling (up-rooting) date. Generally, straw from younger plants (flowering/green capsule stage) rets more quickly than that of more mature plants (yellow/brown capsule stage). This is thought to be related to differences in cell wall composition (e.g., pectin/lignin modifications and/or deposition) and water content (Meijer et al., 1995; Day et al., 2005; Akin, 2013). Our results showing that there was no significant difference in microbial communities and colonization dynamics between the early vs. standard cultures would suggest that differences in retting time may indeed be related to differences in cell wall structure and not to population differences.

Compared to litter decay that normally proceeds undisturbed, dew-retting is a semi-controlled process during which the straw swaths are turned by farmers to obtain a more uniform fiber separation. Our analyses revealed that this practice had a significant effect on both bacterial and fungal community membership and structure of the flax straw microbiome confirming a real microbiological effect of swath turning that probably contributes to a more uniform retting.

Although our results indicated no significant correlation between measured climatic conditions (temperature and rainfall) and community structures during the retting period it is important to remember that our study was conducted within a single year. It is possible that significant variations in community structures may occur between different seasons and further work is necessary to clarify this point.

### Microbial dynamics

During dew-retting the relative abundance of the Bacteroidetes phylum increases while that of the Protobacteria decreases. A similar dynamic also occurs during biodegradation of field biomass from different angiosperm species (e.g., *Arundo donax, Eucalyptus camaldulensis*, and *Populus nigra*) suggesting, as might be expected, that similarities exist between the temporary dew-retting ecosystem and degradation of lignocellulose in the field (Ventorino et al., 2015). Interestingly, the bacterial dynamics of flax dew-retting appear to be closer to that of field lignocellulose degradation than to that observed during flax water retting where Protobacteria increased during retting (Zhao et al., 2016). In this latter case, the phylum Proteobacteria was mainly represented by the genera Azotobacter that increased during retting and (to a much lesser extent) Enterobacter that remained constant. For fungal phyla we observed an increase in the relative abundance of Ascomycota at the expense of Basidiomycota in contrast to the situation generally observed during both field and forest litter decomposition (Schneider et al., 2012; Kuramae et al., 2013; Voříšková and Baldrian, 2013). The observed increase of Ascomycota was due to the saprophytic Altenaria species (Dang et al., 2015) that has previously been linked to later stages of dew-retting (Brown et al., 1986). In contrast, Altenaria species are more abundant during initial stages of litter decay (Snajdr et al., 2011). Our results also indicated that *C. herbarum* and *Epicoccum nigrum* contributed to the increase in Ascomycota relative abundance. During this stage less recalcitrant components of the biomass (pectins, and hemicelluloses) are progressively degraded (Dilly et al., 2001). Contrary to litter decay, dew-retting is a semi-controlled process and the challenge is to limit degradation of major quality related polymers such as crystalline cellulose. In this context, changes in the relative abundance of Ascomycota vs. Basidiomycota could represent an interesting bioindicator of retting progress.

More detailed information on population dynamics at different time points during retting was provided by analyzing the relative abundance of OTUs at different taxonomic rank (e.g., phyla, classes, or genus/species level). The most abundant bacterial OTU corresponded to Sphingomonas sp. that was present throughout most of the retting period in both early and standard cultures. Although Sphingomonas species have been previously identified during bamboo and hemp retting, as well as in forest litter microbiome, this is the first time they have been found in flax retting (Fu et al., 2011; Ribeiro et al., 2015) (Urbanová et al., 2015). These species are able to hydrolyze terminal non-reducing alpha-L-rhamnose residues in alpha-L-rhamnosides giving them the ability to degrade pectin (rhamnogalacturonan I and rhamnogalacturonan II) in the middle lamella (Hashimoto and Murata, 1998). Another *Sphingomonas* species, *S. paucimobilis* is also able to degrade lignin (Masai et al., 1999; de Gonzalo et al., 2016). The second most abundant OTU corresponded to *P. rhizosphaerae*, present during the early and medium retting stages but decreasing in latter stages. A number of Pseudomonas species have previously been associated with retting of different fiber plants (e.g., flax, hemp, jute, ramie) (Rosemberg, 1965; Munshi and Chattoo, 2008; Duan et al., 2012; Ribeiro et al., 2015). Pseudomonas sp. is considered as one of the most efficient lignin degradation bacterium (Shui Yang et al., 2007) and the genomes of both *Pseudomonas putida* and *Pseudomonas aeruginosa* contain genes encoding endoglucanases (Talia et al., 2012). Other abundant OTUs corresponded to Rhizobium, Pedobacter, and Flavobacterium that are known to show pectinase, cellulose, and hemicellulose activities (Mateos et al., 1992; McBride et al., 2009; López-Mondéjar et al., 2016). In addition, Pedobacter has also been identified during bamboo and hemp retting (Fu et al., 2011; Ribeiro et al., 2015) or forest litter degradation (Urbanová et al., 2015). In contrast to Sphingomonas and Pseudomonas, these organisms become more abundant toward the end of the retting period and could be associated with “over-retting” when the structural integrity of the fiber starts to be degraded.

In contrast to the more evenly distributed abundance of the bacterial OTUs, fungal OTUs were dominated by one major species—*C. herbarum*—that rapidly increased during early retting. This species, as well as the third most abundant OTU (*E. nigrum*) are known to be common dew-retting agents and are believed to degrade cellulose (Brown, 1984). Of the other fungal OTUs, all have previously been associated with dew-/water-retting except for *Itersonilia perplexans*. Interestingly, our results also indicated that *Alternaria alternata* is present at the start of retting. Traditionally, the appearance of this species is used as a signal that retting is starting to go too far and that the swaths should be collected (Brown et al., 1986).

### Hydrolytic enzyme potential

Prediction of hydrolytic enzymes potentially present during retting was performed by using PICRUSt (Langille et al., 2013). This software successfully predicts bacterial enzymatic activities represented in different databases (e.g., KEGG Ortholog, COGs, or CAZy). Overall, a large collection of enzyme activities targeting both the main backbones and side chains of the major polysaccharide polymers were identified. Based on OTU counts, ~38, 43, and 19 percent of the total hydrolytic enzyme potential targeted pectins, hemicelluloses, and cellulose, respectively. Despite the clear dynamics and significant changes in the straw microbiome these values remained constant throughout the retting period. Similar software does not exist for predicting fungal enzyme potential. This represents an important hurdle for obtaining a complete overview of the dew-retting process as fungi are major producers of extracellular hydrolytic enzymes (Schneider et al., 2012). Nevertheless, FUNGuild analysis showed that pathogenic taxa, present at the beginning of retting are progressively replaced by saprophytic fungi, more able to degrade lignocellulose. This change is most likely related to the fact that flax plants are still living when up-rooted.

In conclusion, we have shown that HTS metabarcoding is a powerful technique for analyzing complex bacterial and fungal community dynamics during flax dew-retting that can be used to identify different factors affecting the microbiota and—potentially—fiber isolation and quality. However, these results were obtained on samples retted in 1 year and it will be necessary to validate these data over several seasons. The use of PICRUSt data allows a predictive study of potential bacterial hydrolytic activity but should be coupled in future studies with alternative meta-omics methods such as metatranscriptomic or metaproteomic coupled with metagenomics to facilitate the assembling with appropriate reference genomes (Schneider et al., 2012; Dai et al., 2015; Hesse et al., 2015; Kuske et al., 2015; Wu et al., 2015). Such an approach would not only allow confirmation of bacterial enzyme dynamics but would also enable identification of fungal enzymes involved in this process.

## Availability of data and materials

The microbial DNA sequencing data sets supporting the results in this article are available at the EBI ENA with accession number PRJEB20299.

## Author contributions

Conceptualization: CD, SG, and SH; Methodology: CD, SG, and SH; Experimentation: CD; Bioinformatic and statiscal analysis: CD; Writing—original draft: CD; Writing—Review and editing: SG and SH; Funding Acquisition: SG and SH.

### Conflict of interest statement

The authors declare that the research was conducted in the absence of any commercial or financial relationships that could be construed as a potential conflict of interest.

## Supplementary material

Supplementary Figure 1 Rainfall, daily amplitudes of temperature and moisture, and retting time points at Martainneville during the 2014 dew-retting period. Raw data from the Abbeville station available on the infoclimat website (https://www.infoclimat.fr).

Supplementary Figure 2 Schematic representation of the experimental site at Martainneville during the 2014 dew-retting campaign.

Supplementary Figure 3 Rarefaction curves of observed OTUs in bacterial (16S) soil **(A)** and stem **(B)** samples, defined at a 97% sequence similarity cut-off.

Supplementary Figure 4 Rarefaction curves of observed OTUs in fungal (ITS) soil **(A)** and plant **(B)** samples, defined at a 97% sequence similarity cut-off.

Supplementary Figure 5 Alpha diversity of bacterial **(A–C)** and fungal **(D–F)** communities in soil and plant samples during flax dew-retting. **(A,D)** Richness (Chao1), **(B,E)** Evenness (Heip's), and **(C,F)** Diversity (Inverse Simpson). Different colors indicate the source samples (light brown, soil early harvest; dark brown, soil standard harvest; light green, plant early harvest; dark green, plant standard harvest). Plots show the distribution of results of 20,548 bacterial and 42,436 fungal sequences subsampled from each sample 1,000 times and calculated for the average (error bars for Chao1 and Inverse Simpson correspond to lower and higher bound 95% confidence intervals and for Heip's represent standard deviation).

Supplementary Figure 6 Before- **(A,C)** and after- **(B,D)** swath turning biomarkers for bacteria **(A,B)** and fungi **(C,D)**. Biomarkers were identified by analyzing differential OTUs abundance using Metastats, Indicator and LEfSe.

Supplementary Figure 7 Heatmaps showing the total counts of all CAZyme families in early and standard harvests obtained from the OTUs table (Greengenes Database used for consensus taxonomy) and generated by PICRUSt software.
