# Supplementary material for: Characterization of Bacterial and Fungal Community Dynamics by High-Throughput Sequencing (HTS) Metabarcoding during Flax Dew-Retting
Source: Front Microbiol. 2017 Oct 20;8:2052. doi: 10.3389/fmicb.2017.02052 (PMC5655573; doi:10.3389/fmicb.2017.02052)
Supplement: Supplementary Figure 1 — Rainfall, daily amplitudes of temperature and moisture, and retting time points at Martainneville during the 2014 dew-retting period. Raw data from the Abbeville station available on the infoclimat website (https://www.infoclimat.fr). [file Image1.PDF]

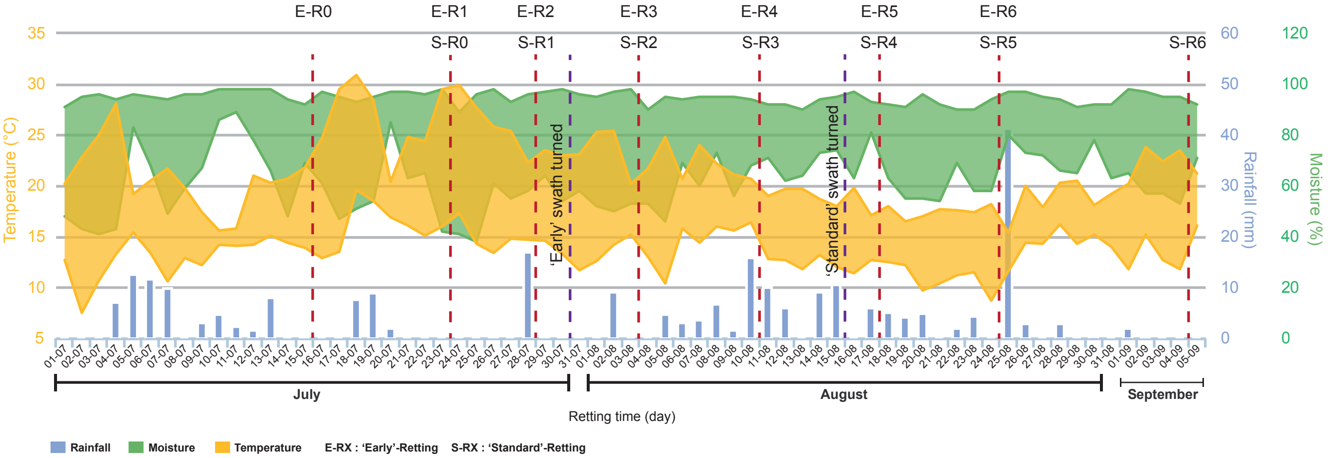

Supplementary Figure 1

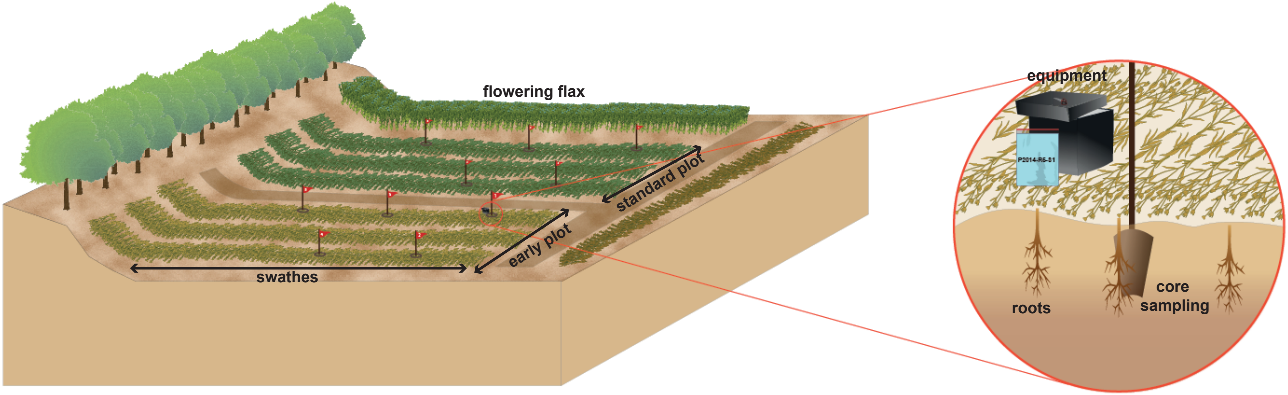

Supplementary Figure 2: Schematic representation of the experimental site at Martainneville during the 2014 dew-retting campaign.

(A)

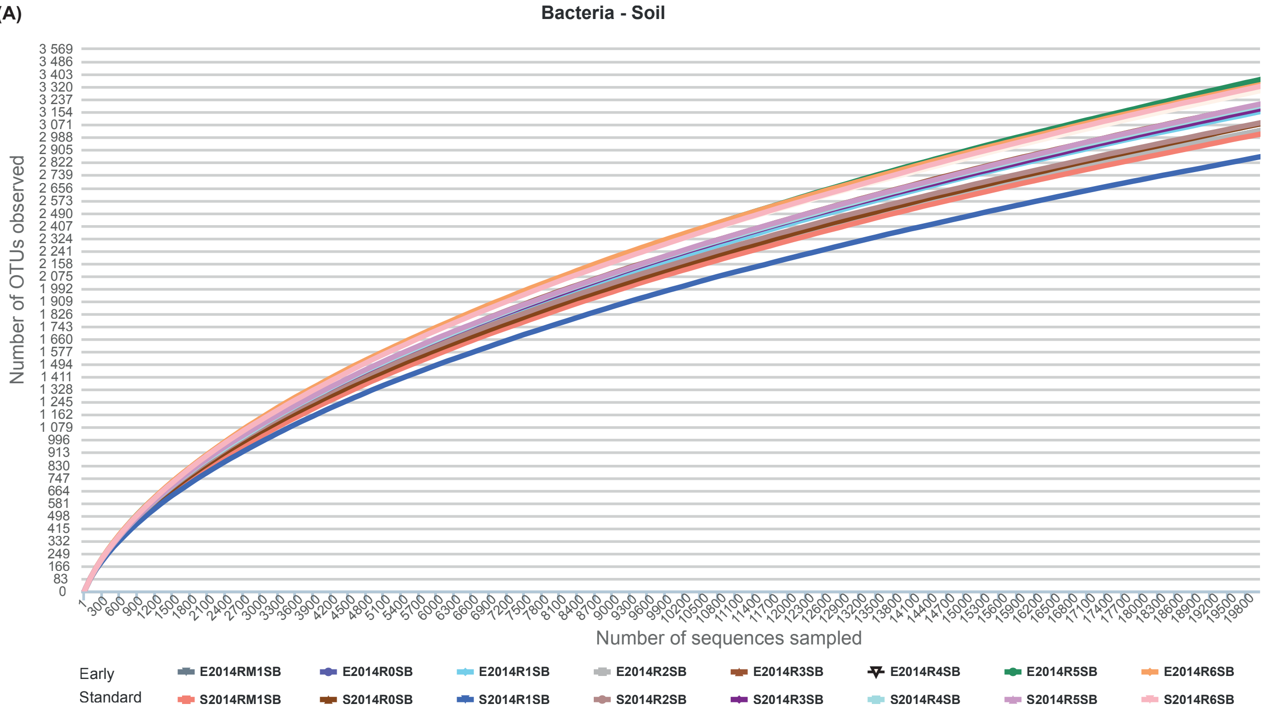

(B)

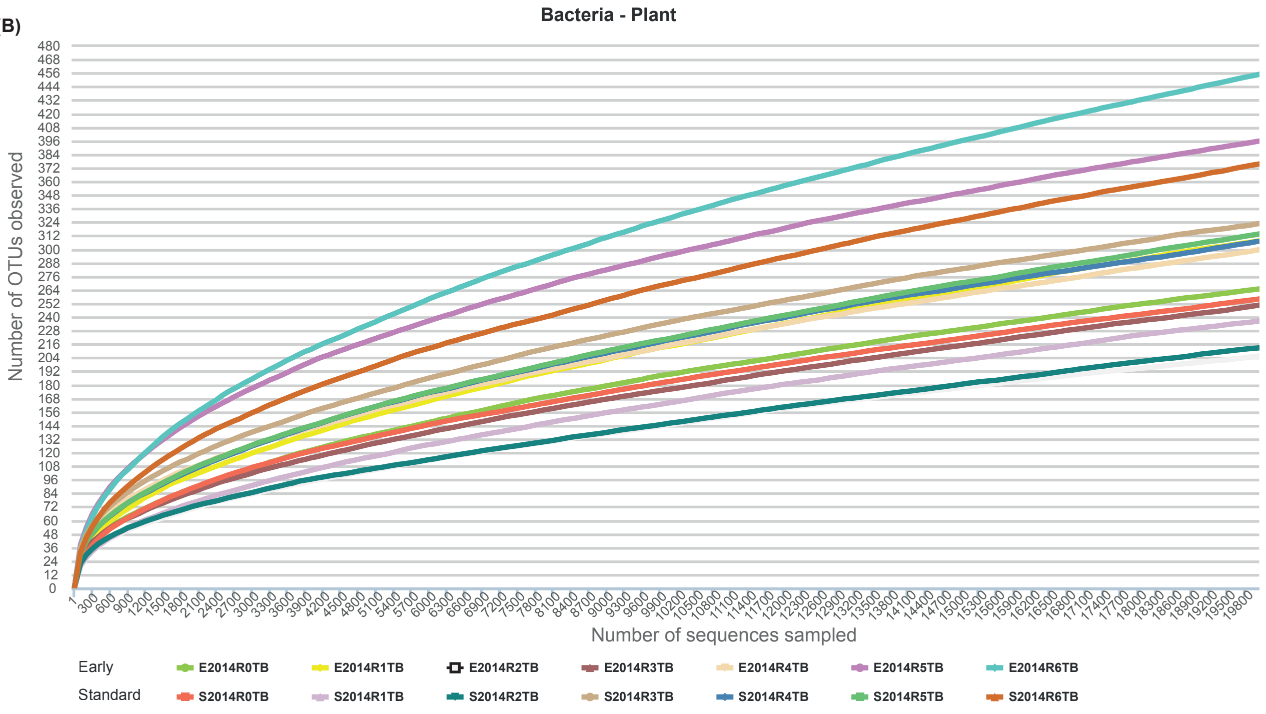

Supplementary Figure 3

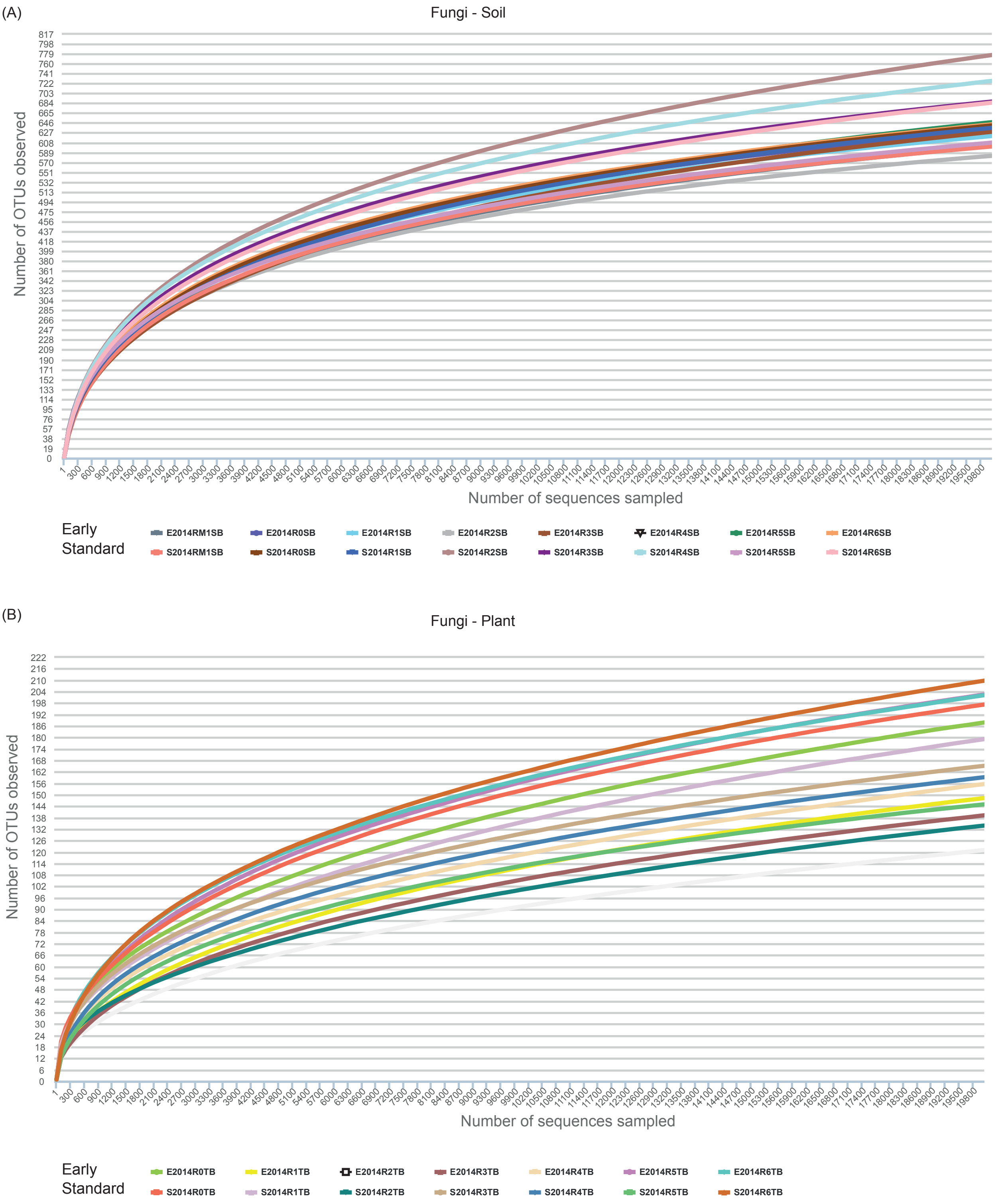

Supplementary Figure 4

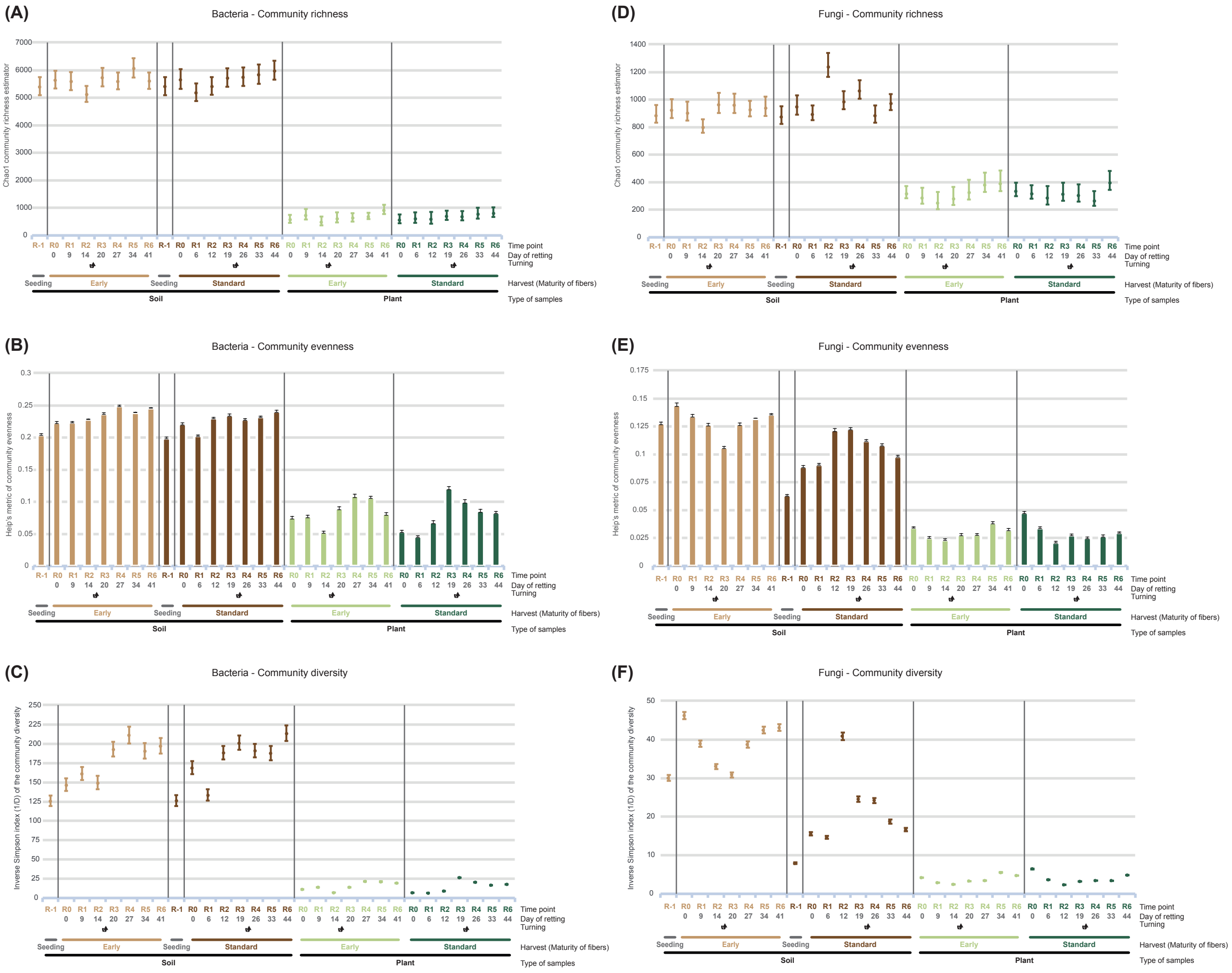

Supplementary Figure 5

(A) Bacteria - Before turning

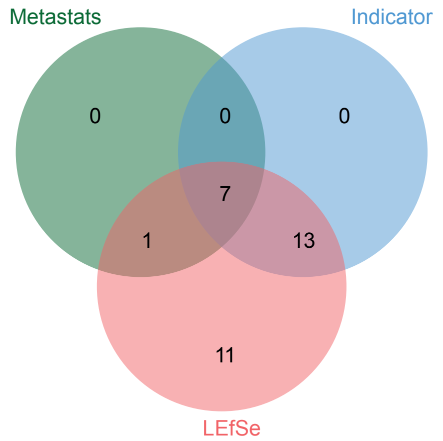

Common elements in Metastats, Indicator and LEfSe:

OTUs	Phyla	Classes	Lowest level of taxonomic assignment
OTU00002	Proteobacteria	Gammaproteobacteria	Pseudomonas sp.
OTU00006	Proteobacteria	Gammaproteobacteria	*Pantoea vagans*
OTU00040	Proteobacteria	Gammaproteobacteria	*Serratia fonticola*
OTU00039	Proteobacteria	Betaproteobacteria	Acidovorax sp.
OTU00016	Proteobacteria	Betaproteobacteria	Duganella sp.
OTU00001	Proteobacteria	Alphaproteobacteria	Sphingomonas sp.
OTU00103	Firmicutes	Bacilli	Paenibacillus sp.

(B) Bacteria - After turning

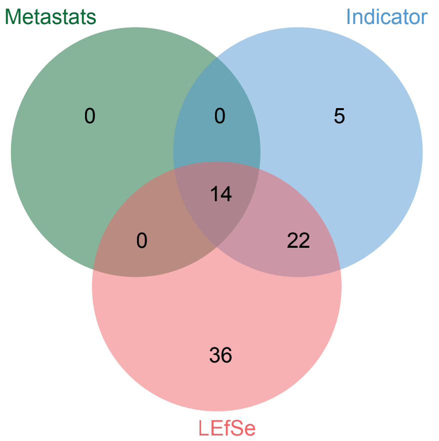

Common elements in Metastats, Indicator and LEfSe:

OTUs	Phyla	Classes	Lowest level of taxonomic assignment
OTU00005	Bacteroidetes	Sphingobacteriia	Pedobacter sp.
OTU00014	Bacteroidetes	Sphingobacteriia	Pedobacter sp.
OTU00026	Bacteroidetes	Flavobacteriia	Flavobacterium sp.
OTU00003	Proteobacteria	Alphaproteobacteria	Rhizobium sp.
OTU00029	Bacteroidetes	Flavobacteriia	Flavobacterium sp.
OTU00019	Proteobacteria	Gammaproteobacteria	Stenotrophomonas sp.
OTU00047	Bacteroidetes	Sphingobacteriia	Mucilaginibacter sp.
OTU00012	Bacteroidetes	Flavobacteriia	Flavobacterium sp.
OTU00013	Bacteroidetes	Flavobacteriia	Chryseobacterium sp.
OTU00041	Proteobacteria	Alphaproteobacteria	Falsirhodobacter sp.
OTU00009	Bacteroidetes	Flavobacteriia	Epilithonimonas sp.
OTU00021	Proteobacteria	Alphaproteobacteria	Brevundimonas sp.
OTU00030	Bacteroidetes	Cytophagia	Dyadobacter sp.

(C) Fungi - Before turning

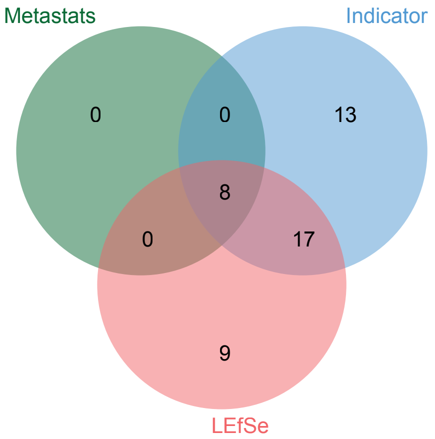

Common elements in Metastats, Indicator and LEfSe:

OTUs	Phyla	Classes	Lowest level of taxonomic assignment
OTU1658	Ascomycota	Sordariomycetes	Microdochium sp.
OTU288	Basidiomycota	Tremellomycetes	*Cryptococcus chernovii* (SH197624.07FU)
OTU1480	Basidiomycota	Tremellomycetes	*Bulleromyces albus* (SH215453.07FU)
OTU448	Basidiomycota	Tremellomycetes	*Itersonilia perplexans* (SH199072.07FU)
OTU3168	Ascomycota	Dothideomycetes	Septoria sp.
OTU2164	Ascomycota	Dothideomycetes	*Ascochyta skagwayensis* (SH215821.07FU)
OTU1841	Ascomycota	Sordariomycetes	*Monographella nivalis* (SH213511.07FU)
OTU945	Basidiomycota	Microbotryomycetes	*Sporobolomyces roseus*

(D) Fungi - After turning

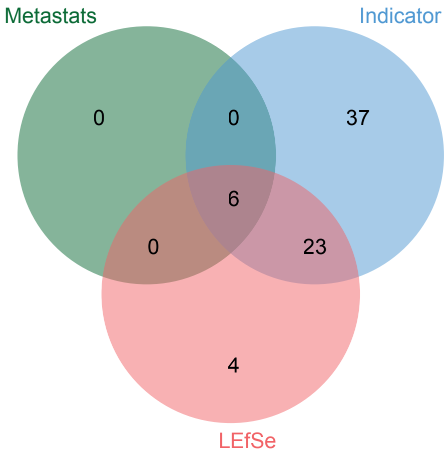

Common elements in Metastats, Indicator and LEfSe:

OTUs	Phyla	Classes	Lowest level of taxonomic assignment
OTU1949	Ascomycota	Dothideomycetes	*Alternaria alternata*
OTU1100	Basidiomycota	Agaricostilbomycetes	Kondoa sp. (SH476649.07FU)
OTU2906	Ascomycota	Dothideomycetes	Cladosporium sp.
OTU2206	Ascomycota	Dothideomycetes	*Epicoccum nigrum*
OTU1918	Ascomycota	Dothideomycetes	Pleosporaceae

Supplementary Figure 6

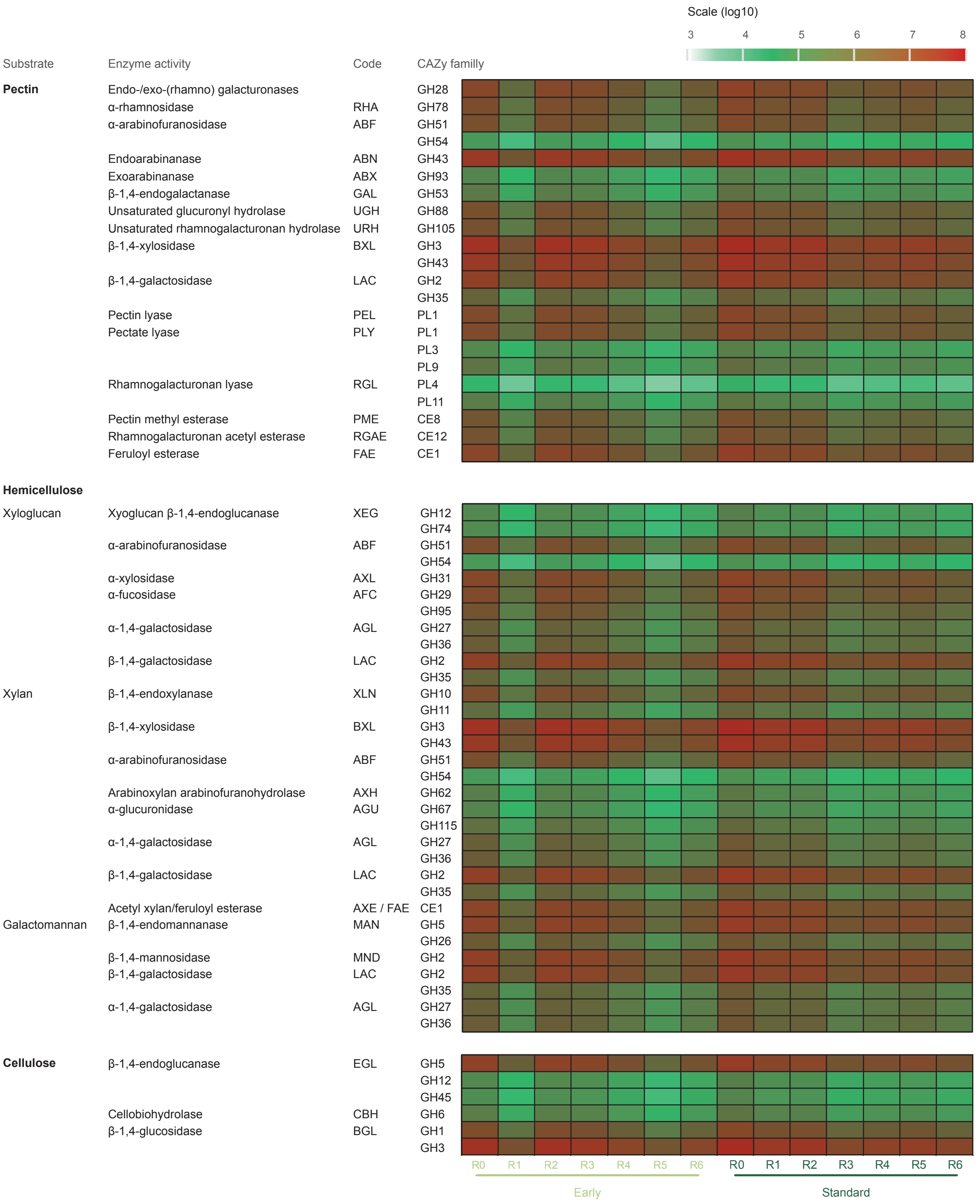

Supplementary Figure 7
